# A five‐miRNA panel in plasma was identified for breast cancer diagnosis

**DOI:** 10.1002/cam4.2572

**Published:** 2019-09-30

**Authors:** Minghui Li, Xuan Zou, Tiansong Xia, Tongshan Wang, Ping Liu, Xin Zhou, Shui Wang, Wei Zhu

**Affiliations:** ^1^ Department of Breast Surgery First Affiliated Hospital of Nanjing Medical University Nanjing PR China; ^2^ Fudan University Shanghai Cancer Center Shanghai PR China; ^3^ Department of Oncology First Affiliated Hospital of Nanjing Medical University Nanjing PR China; ^4^ Department of Oncology and Radiotherapy Nanjing Pukou Central Hospital Nanjing PR China

**Keywords:** biomarker, breast cancer, diagnosis, plasma miRNA, qRT‐PCR

## Abstract

Breast cancer (BC) is one of the most common cancers in females. Since early detection can bring prognosis benefit, discovery of novel noninvasive biomarkers for BC diagnosis is in urgent need. In this four‐phase study, we profiled miRNA expression in plasma samples from a total of 257 BC patients and 257 normal controls (NCs). Exiqon miRNA qPCR panel was used to select candidate miRNAs in the screening phase which were further analyzed using qRT‐PCR in the following training, testing and external validation phases. Finally, we identified five plasma miRNAs (let‐7b‐5p, miR‐122‐5p, miR‐146b‐5p, miR‐210‐3p and miR‐215‐5p) whose expression levels were significantly different between BC patients and NCs. A 5‐miRNA panel in plasma with high sensitivity and specificity was then constructed to detect BC. The areas under the receiver‐operating characteristic curves (AUCs) of the panel were 0.683, 0.966, 0.978 for the training, testing and external validation sets, respectively. Expression of the identified miRNAs was further analyzed among 32 pairs of BC tissue and the adjacent normal tissue samples as well as plasma‐derived exosome samples from 32 BC patients vs 32 NCs. Let‐7b‐5p was contrarily down‐regulated in BC tissue. In exosomes samples, only miR‐122‐5p was significantly up‐regulated as in plasma for BC patients. In conclusion, we identified a 5‐miRNA plasma panel (let‐7b‐5p, miR‐122‐5p, miR‐146b‐5p, miR‐210‐3p and miR‐215‐5p) that could serve as a promising biomarker for BC detection.

## Graphical abstract

We identified five plasma miRNAs (let‐7b‐5p, miR‐122‐5p, miR‐146b‐5p, miR‐210‐3p and miR‐215‐5p) by multiple‐phase validation which could serve as novel noninvasive biomarkers for BC diagnosis. Our findings may contribute to the early detection of BC and be beneficial to BC patients.

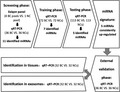

## 1 INTRODUCTION

Breast cancer (BC) is a great health threat to females worldwide. The profile of the most commonly diagnosed cancers across countries from GLOBOCAN 2018 shows that BC is the most frequently diagnosed cancer type in the vast majority of countries (154 of 185, including China). In Chinese women, BC is the second leading cause of death just after lung cancer.^1^ Though the incidence and death rate of BC has gradually decreased in recently years in America, it is still a heavy health burden in China compared to high‐income countries, probably due to lower rates of provision, delays in diagnosis, inadequate medical resources, lack of awareness and other factors reflecting the socioeconomic gap.^2^, ^3^, ^4^ With the rapid development of medical technology, strategies such as surgery, medication and radiotherapy can help a lot to reduce mortality rate.^5^ Though clinical intervention at early stage can greatly improve prognosis, many BC patients are asymptomatic until disease progression.^6^ Thus, effective screening methods are in great demand for the early detection of BC. In clinical practice, many screening strategies have been widely used, such as mammography, breast magnetic resonance imaging (MRI) and ultrasound imaging.^7^, ^8^ However, these strategies are far from being perfect because of over‐diagnosis, false‐positive, inconsistent results and potential radiation injury.^9^, ^10^, ^11^ Core needle aspiration can help establish the diagnosis, but the procedure is invasive and not suitable for routine use.^12^ Therefore, novel non‐invasive screening methods of high sensitivity and specificity are needed to assist the early diagnosis of BC.

MicroRNAs (miRNAs), families of small noncoding RNAs, are important posttranscriptional regulators of gene expression.^13^ Numerous studies have shown that miRNAs played important roles in nearly all biological processes and their aberrant expression was associated with many diseases including cancers.^14^ Stable existence of miRNAs in peripheral blood circulation discovered by Mitchell et al revealed that circulating miRNAs could be promising noninvasive biomarkers for cancer detection.^15^ For BC, more and more circulating miRNAs (ie, miR‐155 and miR‐21) are emerging as potential diagnostic or prognostic biomarkers.^16^, ^17^, ^18^ However, these findings often differed from each other due to different experiment design, study cohorts or disease status.^19^ Thus, much larger prospective studies need to be conducted for the discovery of valid miRNA biomarkers with higher sensitivity and specificity for BC detection. In this study, we focused on plasma miRNAs and designed a controlled experiment based on Exiqon miRNA qPCR panel and quantitative reverse transcription polymerase chain reaction (qRT‐PCR) which was divided into four phases. MiRNA expression in plasma exosomes and tissue samples was also analyzed for better understanding of possible mechanisms.

## 2 MATERIALS AND METHODS

### 2.1 Study subjects, samples and study design

A total of 546 females (289 histopathologically diagnosed BC patients and 257 normal controls (NCs) who underwent routine health checkup) were recruited in this study. They were all participants from the First Affiliated Hospital of Nanjing Medical University during 2014 and 2016. Clinical characteristics of each patient were recorded. The experiment was approved by the institutional ethics committee and written informed consent was obtained from each participant in advance.

Whole blood samples (5 mL) were collected with ethylenediaminetetraacetic acid (EDTA)‐containing tubes from healthy donors and BC patients before they received any clinical intervention. Plasma was separated from whole blood within 12 hours following a two‐step centrifugal protocol: 350 RCF (reactive centrifugal force) for 10 minutes and 20 000 RCF for 10 minutes (Beckman Coulter). The obtained plasma samples were stored at −80°C until use.

**Figure 1:**
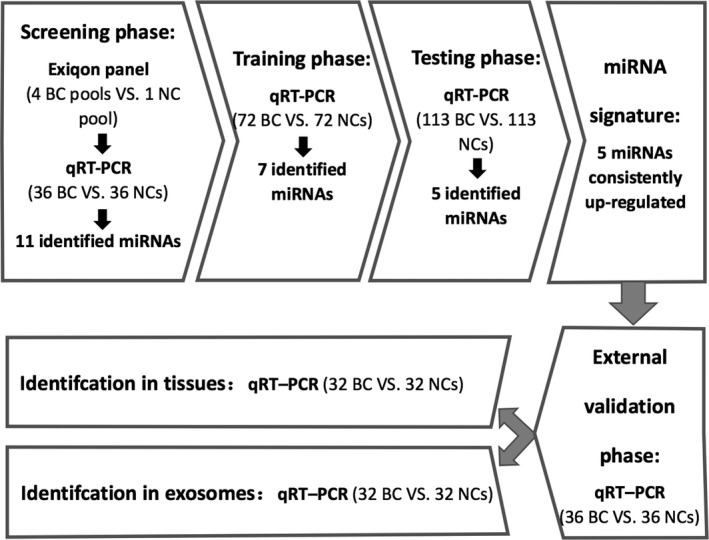
The flow chart of study design. BC, breast cancer; NC, normal control; qRT‐PCR, quantitative reverse transcription polymerase chain reaction

In all, we collected 257 BC and 257 NCs plasma samples which were divided into four sets and analyzed in four independent phases in sequence (Figure 1): the screening phase (36 BC vs 36 NCs), training (72 BC vs 72 NCs), testing (113 BC vs 113 NCs) and external validation phase (36 BC vs 36 NCs). In the screening phase, we conducted a two‐step screen for selection of candidate miRNAs: (I) application of Exiqon miRCURY‐Ready‐to‐Use PCR‐Human‐panel‐I+II‐ V1.M (Exiqon miRNA qPCR panel; 179 miRNAs) based on pooled plasma samples (II) further confirmation using qRT‐PCR based on additional plasma samples (36 BC vs 36 NCs). In step‐one, we chose 40 plasma samples from BC patients and 10 plasma samples from healthy controls. The 40 BC samples included 10 from HER2+, HR‐ (ER‐ and PR‐) patients, 10 from HER2‐, HR+ (ER+ or PR+) patients, 10 from HER2‐, HR‐ (triple‐negative) patients and another 10 samples from randomly selected BC patients without consideration of their molecular subtyping. Each of the 10 samples were pooled as one pool sample to reduce the impact of variation of each individual sample in the results. Approximately 20‐25 ng RNA was isolated from each of the four BC pools and one NC pool for miRNA microarrays. Each BC pool sample was compared to NC to obtain a list of differentially expressed miRNAs. The result of candidate miRNAs was the union of the four separate lists, which means that if expression of miRNA was of significant difference in any one of the four BC pool samples, it could be further validated in additional 36 BC and 36 NC plasma samples using qRT‐PCR before entering into the next training and testing phase. Finally, identified miRNAs from the previous three phases were verified in the external validation phase to consolidate the findings.

In addition, 32 pairs of BC tissue and adjacent normal tissue specimens were collected from 32 BC patients undergoing surgical operation to decipher miRNA expression using qRT‐PCR assays. The obtained tissue samples were kept in liquid nitrogen until further use. MiRNAs from plasma‐derived exosomes were also explored in 32 BC patients and 32 NCs.

### 2.2 Exosomes isolation

We used Exo‐Quick Exosome Precipitation Solution (System Biosciences) to isolate exosomes from plasma samples. According to the manufacturer's protocol, exosome pellets were precipitated from the mixture of 200 μL plasma and 50 μL ExoQuick exosome precipitation solution and dissolved in 200 μL RNase‐free water for further RNA extraction.

### 2.3 RNA extraction

For plasma and exosome samples, total RNA was isolated using the mirVana PARIS Kit (Ambion) following the manufacturer's protocol. After the addition of denaturing solution (Ambion), 5 μL synthetic *C elegans* miRNA *cel‐miR‐39* (5 nM/L, RiboBio) was added to each sample to normalize sample‐to‐sample variation. For tissue specimens, we used TRIzol (Invitrogen) to extract total RNA in accordance with the manufacturer's instruction. The acquired total RNA was finally eluted into 100 μL of RNase‐free water and kept at −80°C until further use. The concentration and purity of total RNA was measured using a Nanodrop 2000 spectrophotometer (NanoDrop Technologies). Samples with total RNA concentration <10 ng/μL were excluded in the analysis.

### 2.4 Quantitative reverse transcription polymerase chain reaction (qRT‐PCR)

MiRNAs were amplified using a Bulge‐Loop^TM^ miRNA qRT‐PCR Primer Set (RiboBio) which contained specific primers for reverse transcription (RT) and polymerase chain reaction (PCR). The qRT‐PCR was run in triplicate on a LightCycler^®^ 480 Real‐Time PCR System (Roche Diagnostics) in 384‐well plates. As described previously,^20^ the RT reaction was performed at 42°C for 60 minutes and then 70°C for 10 minutes; the following PCR reaction was performed at 95°C for 20 seconds, followed by 40 cycles of 95°C for 10 seconds and 60°C for 20 seconds, and then 70°C for 10 seconds. The amount of PCR products was evaluated based on the level of fluorescence emitted by SYBR Green (SYBR^®^ Premix Ex Taq^TM^ II, TaKaRa). The specificity of PCR products was assessed using melting curve analysis. The combination of exogenous reference miRNA (*cel‐miR‐39*) and endogenous reference miRNA (*miR‐16* for plasma and exosomes samples; *RNU6B* (*U6*) for tissue samples) was introduced into data analysis procedure for normalization. The selection criteria of reference miRNAs is consistent with previous studies.^15^, ^21^ The relative expression level of each miRNA was determined using the 2^−ΔΔCt^ method (ΔCt = Ct_miRNA_−1/2 (Ct*_cel‐miR‐39_* + Ct_endogenous reference miRNA_); Ct: the threshold cycle).^22^

### 2.5 Statistical analysis

The difference of miRNA expression level in plasma and exosomes between cases and controls was determined using Mann‐Whitney *U* test, and Wilcoxon test was applied for the comparison between paired tissue samples. A binary logistic regression model was constructed to establish the miRNA panel. The diagnostic value of identified miRNA was evaluated based on receiver‐operating characteristic (ROC) curve and area under the ROC curve (AUC). All statistical analysis was performed using SPSS20.0 software (SPSS Inc) and GraphPad Prism 7.0 (GraphPad Software). A two‐sided *P* < .05 was considered to be statistically significant.

## 3 RESULTS

### 3.1 Characteristics of subjects

Plasma samples from 257 BC patients and 257 NCs were randomly divided into four parts: 36 BC vs 36 NCs for validation using qRT‐PCR in the screening phase, 72 BC vs 72 NCs, 113 BC vs 113 NCs and 36 BC vs 36 NCs for the training, testing and external validation phases, respectively. The flow chart of the study design is shown in Figure 1. The demographics and clinical characteristics of BC patients and NCs are listed in Table 1. There was no significant difference in age distribution between the two groups in any phase (*P* > .05).

**Table 1:** Demographic and clinical characteristics of the participants in the study

Characteristics	Screening set		Training set		Testing set		External validation set	
	BC patients (%)	HCs (%)	BC patients (%)	HCs (%)	BC patients (%)	HCs (%)	BC patients (%)	HCs (%)
Number	36	36	72	72	113	113	36	36
Age at diagnosis								
<50	13 (36.1)	16 (44.4)	28 (38.9)	33 (45.8)	43 (38.1)	37 (32.7)	15 (41.7)	14 (38.9)
≥50	23 (63.9)	20 (55.6)	44 (61.1)	39 (54.2)	70 (61.9)	76 (67.3)	21 (58.3)	22 (61.1)
TNM stage								
In situ	2 (5.6)		10 (13.9)		5 (4.4)		2 (5.6)	
I	12 (33.3)		14 (19.4)		32 (28.3)		10 (27.8)	
II	16 (44.4)		36 (50.0)		55 (48.7)		12 (33.3)	
III	6 (16.7)		12 (16.7)		21 (18.6)		12 (33.3)	
Grade								
I	3 (8.3)		3 (4.2)		4 (3.5)		2 (5.6)	
II	11 (30.6)		27 (37.5)		46 (40.7)		15 (41.7)	
III	22 (61.1)		42 (58.3)		63 (55.8)		19 (52.8)	
Epithelial subtype								
Luminal	10 (27.8)		28 (38.9)		53 (46.9)		22 (61.1)	
HER2‐enriched	10 (27.8)		18 (25.0)		21 (18.6)		6 (16.7)	
Triple‐negative	14 (38.9)		16 (22.2)		34 (30.1)		6 (16.7)	
In situ	2 (5.6)		10 (13.9)		5 (4.4)		2 (5.6)	

### 3.2 Selection of candidate miRNAs from pooled plasma samples

In the screening phase, miRCURY‐Ready‐to‐Use PCR‐Human‐panel‐I+II‐V1.M based on the qRT‐PCR platform was applied to find candidate miRNAs which were differently expressed between any of the four BC pooled samples and one NC pooled sample. A total of 178 miRNAs with high abundance in peripheral plasma/serum were sequenced. MiRNAs with Ct‐value less than 37 and five lower than negative control (No Template Control, NTC) were included in data analysis. An miRNA was considered to be a candidate miRNA if its expression level was altered with a fold change (FC)> 1.5 or <0.67 in BC pools compared to NC pool. As a result, 29 miRNAs (21 up‐regulated and 8 down‐regulated) were initially selected, and their expression levels were further analyzed in another cohorts of plasma samples (36 BC VS. 36 NCs) using qRT‐PCR. In the end, 11 miRNAs (let‐7b‐5p, miR‐122‐5p, miR‐151a‐3p, miR‐215‐5p, miR‐223‐5p, miR‐23a‐3p, miR‐660‐5p, miR‐126‐5p, miR‐146b‐5p, miR‐210‐3p and miR‐222‐3p) which were consistently up‐regulated (FC > 1.5) in both of the screening steps were chosen to be further analyzed in the following phases (Table S1). Notably, all of the eight down‐regulated miRNAs were left out in this step. We supposed that the expression of these miRNAs might be quite limited in peripheral plasma, and thus lacked value and stability as a biomarker.

### 3.3 Confirmation of candidate miRNAs using qRT‐PCR

**Figure 2:**
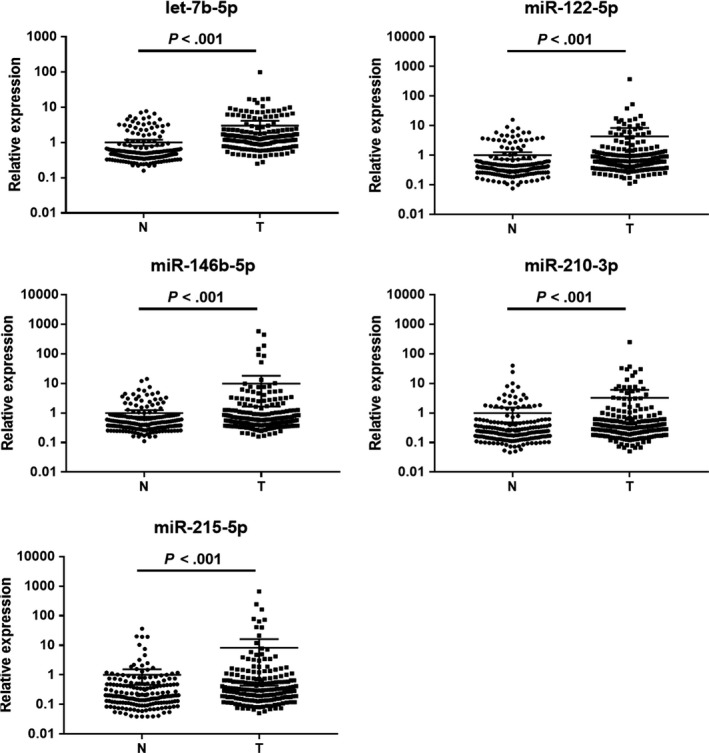
Expression levels of the identified miRNAs in plasma among 185 BC patients and 185 NCs in the combination of training and testing sets. N, normal control; T, tumor; Horizontal line, mean with 95% CI

The expression of the 11 candidate miRNAs from the screening phase was further analyzed in 72 BC patients and 72 NCs using qRT‐PCR in the training phase. Seven miRNAs (let‐7b‐5p, miR‐122‐5p, miR‐146b‐5p, miR‐210‐3p, miR‐215‐5p, miR‐222‐3p and miR‐660‐5p) out of the 11 were still up‐regulated in BC plasma samples compared to NCs (*P* < .05). These miRNAs were further explored in the larger cohort of 113 BC patients and 113 NCs using qRT‐PCR in the testing phase. As a result, only five miRNAs including let‐7b‐5p, miR‐122‐5p, miR‐146b‐5p, miR‐210‐3p and miR‐215‐5p showed a consistent trend of up‐regulation in BC plasma samples in the testing set as in the training set (FC > 1.5; *P* < .05). When the training and testing sets were combined, let‐7b‐5p, miR‐122‐5p, miR‐146b‐5p, miR‐210‐3p and miR‐215‐5p were all significantly up‐regulated in BC plasma samples compared to NCs (*P* < .05; Figure 2; Table 2; the other miRNAs not passing through the two phases are shown in Table S2).

**Table 2:** Expression levels of the five plasma miRNAs in the training and testing sets; presented as mean ± SD

miRNA	Training set				Testing set				Combined		
	BC	HC	FC	*P* value	BC	HC	FC	*P* value	FDR	FC	*P* value
let‐7b‐5p	3.82 ± 2.01	4.81 ± 1.44	1.98	.001	4.52 ± 0.81	6.11 ± 0.63	3.03	<.001	<0.001	2.56	<.001
miR‐122‐5p	4.00 ± 2.44	4.86 ± 1.61	1.82	.042	5.42 ± 1.12	6.50 ± 0.68	2.11	<.001	<0.001	1.99	<.001
miR‐146b‐5p	8.45 ± 2.62	9.47 ± 1.37	2.02	.045	10.34 ± 0.92	10.99 ± 0.63	1.57	<.001	<0.001	1.73	<.001
miR‐210‐3p	4.63 ± 2.77	5.63 ± 2.05	2.01	.036	6.44 ± 1.18	7.03 ± 0.92	1.50	<.001	<0.001	1.68	<.001
miR‐215‐5p	3.95 ± 3.19	5.16 ± 2.15	2.31	.042	5.69 ± 1.36	6.49 ± 1.11	1.74	<.001	<0.001	1.94	<.001

Abbreviations: ΔCT, relative to combination of cel‐miR‐39 and miR‐16; FC, fold change; FDR, false discovery rate.

### 3.4 Diagnostic value of identified miRNA signature in plasma

**Figure 3:**
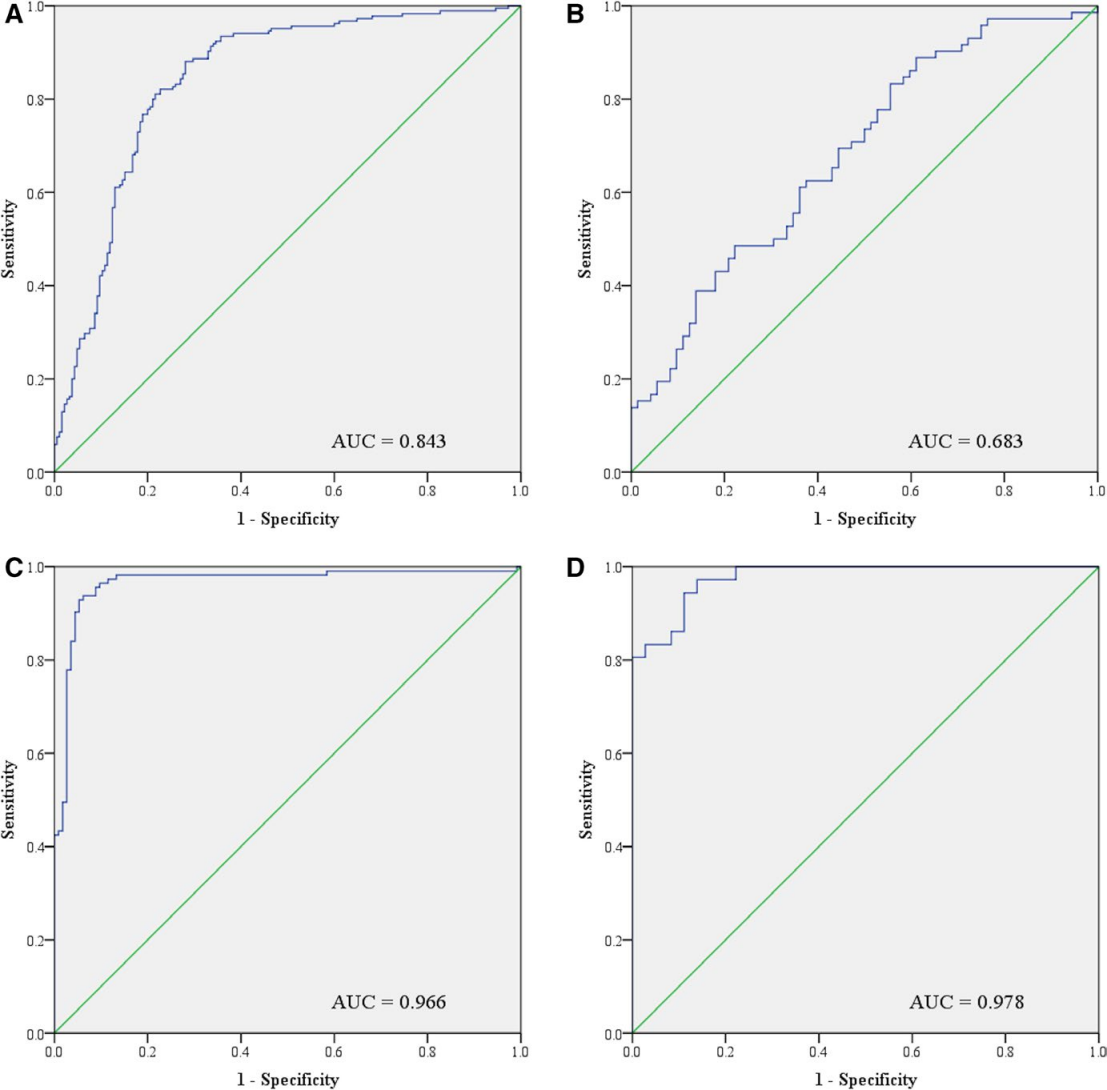
ROC curve analysis of the five‐miRNA panel for BC diagnosis. A. Combined training and testing sets (185 BC vs 185 NCs); B. training set (72 BC vs 72 NCs); C. testing set (113 BC vs 113 NCs); D. external validation set (36 BC vs 36 NCs). AUC, area under the ROC curve; ROC curve, receiver‐operating characteristic curve

ROC curve analysis was performed to evaluate the diagnostic value of the five identified miRNAs for BC. We combined the five miRNAs together and constructed a 5‐miRNA panel for BC diagnosis. The predicted probability of BC detection from the logistic regression model was calculated using the formula: Logit(P) = 3.059 − 1.651 × let‐7b‐5p – 0.212 × miR‐122‐5p + 0.147 × miR‐146b‐5p + 0.938 × miR‐210‐3p – 0.169 × miR‐215‐5p. The AUCs for the 5‐miRNA plasma signature were 0.683 (95% confidence interval (CI): 0.597‐0.769; Figure 3B) for the training set and 0.966 (95% CI: 0.940‐0.992; Figure 3C) for the testing set, respectively (Table 3). Since relatively smaller sample size may lead to bias of results, independent multi‐stage experiments and larger sample verification can reduce the risk to some extent. This study adopted the strategy of multi‐phase verification. Then, in order to evaluate the research result among a larger sample, we also combined the data of training and testing sets for combined estimation. As a result, the AUCs for individual let‐7b‐5p, miR‐122‐5p, miR‐146b‐5p, miR‐210‐3p and miR‐215‐5p were 0.808 (95% CI: 0.761‐0.855), 0.687 (95% CI: 0.632‐0.741), 0.623 (95% CI: 0.566‐0.679), 0.613 (95% CI: 0.556‐0.670) and 0.620 (95% CI: 0.563‐0.677), respectively (Figure S1). The AUC for the panel was 0.843 (95% CI: 0.801‐0.884; Figure 3A) in the combined training and testing sets (Table 3), relatively higher than each of the five miRNAs, which further indicated better diagnostic ability of the panel than single miRNA.

**Table 3:** Diagnostic value of the 5‐miRNA panel using ROC curves and AUC analysis

Set	AUC value (95% CI)	Sensitivity %	Specificity %
Training set	0.683 (0.597‐0.769)	62.5	61.1
Testing set	0.966 (0.940‐0.992)	93.8	93.8
Training and testing set	0.843 (0.801‐0.884)	81.1	78.4
External validation set	0.978 (0.953‐1.000)	94.4	88.9

Abbreviations: AUC, area under the ROC curve; ROC curve, receiver‐operating characteristic curve.

To verify the diagnostic performance of the panel, an additional cohort of 36 BC patients and 36 NCs was analyzed in the external validation phase. Trend of expression difference between BC patients and NCs remained the same for each of the five miRNAs as the previous phases with *P* < .05 (Table S3), while the AUC was 0.978 (95% CI: 0.953‐1.000; Figure 3D; Table 3) in this phase, which confirmed the diagnostic value of the 5‐miRNA panel in plasma for BC detection.

Moreover, association of the identified miRNAs with clinical parameters including TNM stage, tumor grade and epithelial subtype was also assessed among all the subjects. But no significant difference was observed between the identified miRNAs or the 5‐miRNA panel and any of these clinical characteristics (*P* > .05 (Kruskal‐Wallis rank test or *χ* ^2^ test); data not shown).

### 3.5 miRNA expression in tissue samples

**Figure 4:**
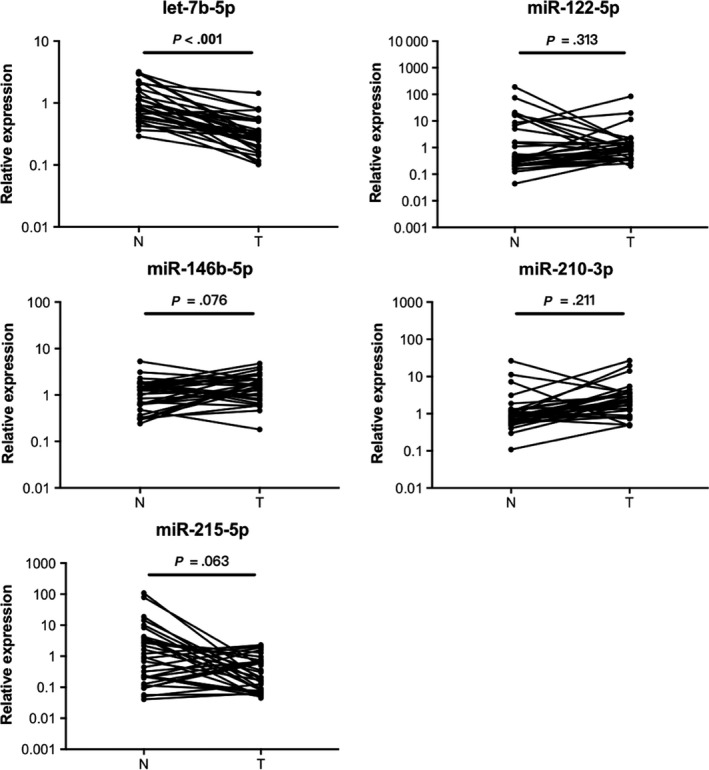
Expression levels of the identified five miRNAs in 32 pairs of BC tissue and the adjacent normal tissue samples. Horizontal line, mean with SEM; N, normal control; T, tumor

The expression levels of the five miRNAs were further analyzed in 32 pairs of BC tumor tissues and the matched adjacent normal tissues. As can be seen in Figure 4, only let‐7b‐5p was significantly down‐regulated in BC tumor tissue samples compared to NCs (*P* < .05). For the expression of the other four identified plasma miRNAs, no significant difference was observed (*P* > .05).

### 3.6 Exploration of miRNAs in plasma exosomes

**Figure 5:**
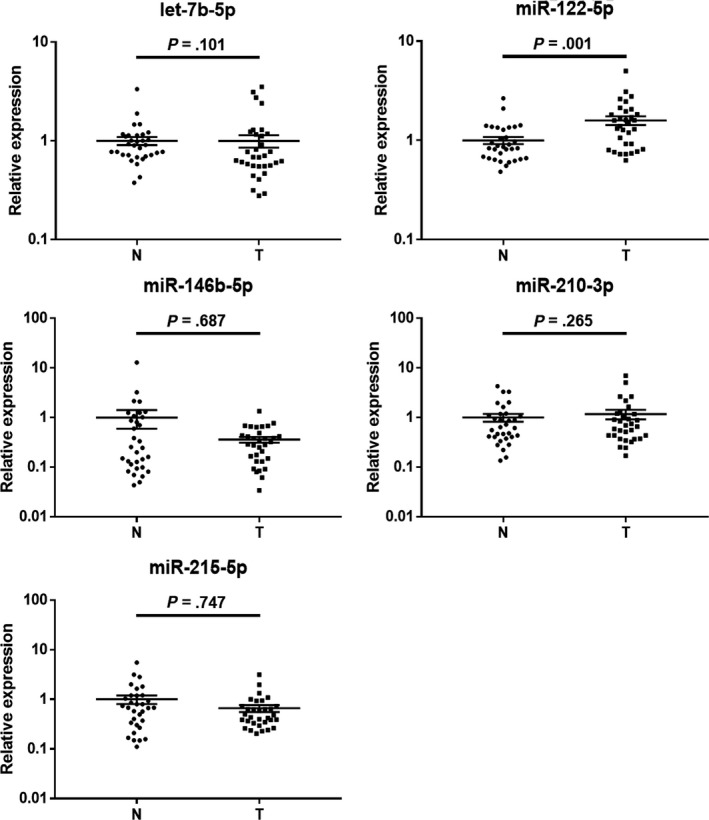
Expression levels of the identified five miRNAs in plasma‐derived exosomes (32 BC vs 32 NCs). Horizontal line, mean with SEM; N, normal control; T, tumor

MiRNA expression was further analyzed in plasma exosomes (32 BC vs 32 NCs) for the exploration of potential forms and function of the identified peripheral miRNAs. Among the five miRNAs, only miR‐122‐5p was significantly up‐regulated in BC plasma‐derived exosomes compared to NCs (*P* < .05; Figure 5).

### 3.7 Bioinformatics analysis of identified miRNAs

**Figure 6:**
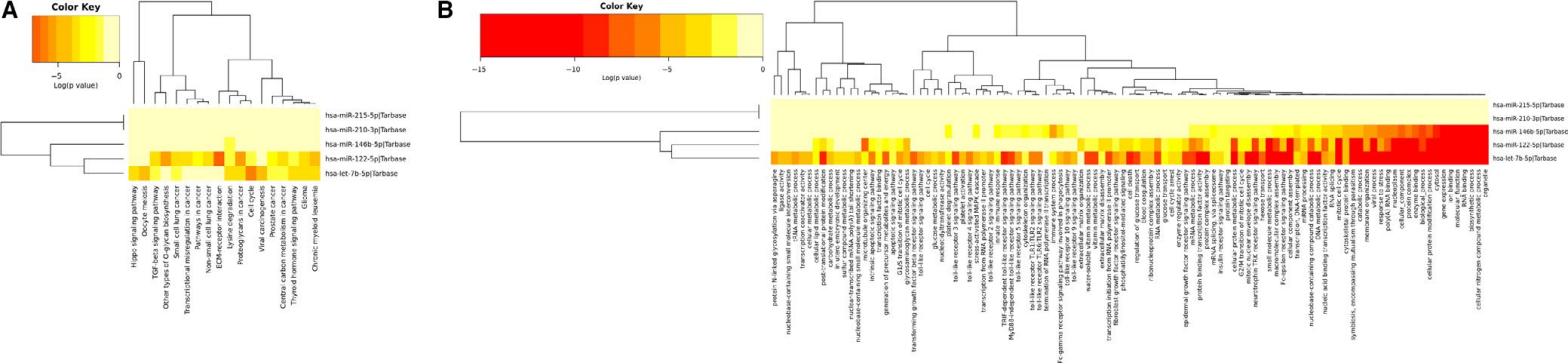
Heatmaps of pathway analysis using KEGG (A) and GO (B) analyses. GO: Gene Ontology; KEGG: Kyoto Encyclopedia of Genes and Genomes

DIANA‐miRPath v3.0, an online miRNA pathway analysis web‐server (http://www.microrna.gr/miRPathv3), was used to predict potential targets of the five identified miRNAs based on experimentally verified miRNA interactions from DIANA‐TarBase7.0. Kyoto Encyclopedia of Genes and Genomes (KEGG) and Gene Ontology (GO) analyses were performed and the heatmaps are given in Figure 6. Several cancer‐related pathways such as transcriptional misregulation in cancer, cell death, cell cycle and epidermal growth factor receptor signaling pathway were found to be associated with some of these identified miRNAs, which indicated the possible roles of these miRNAs in the biological processes of BC.

## 4 DISCUSSION

We designed a four‐phase study (including the screening, training, testing and the external validation phase) using a total of 257 BC and 257 NC plasma samples for the discovery of promising plasma miRNA biomarkers for BC diagnosis.

First, the Exiqon miRNA qPCR panel was used to select candidate miRNAs among four pooled BC plasma samples and one NC pool in the initial screening phase. In BC, different molecular subtypes may have totally different molecular properties, and can indicate different pathological features, therapeutic reactions and clinical prognosis. Expression of ER, PR, HER2 are the most recognized indicators.^23^ Since bias in molecular typing among BC samples might have unknown confounding effects, we specially constructed three pooled samples (1 HER2+HR‐, 1 HER2‐HR+, 1 HER2‐HR‐) with only one random sample rather than obtain plasma pool samples completed by random choice. MiRCURY‐Ready‐to‐Use PCR‐Human–panel‐I+II V1.M (Exiqon) applied in this phase is one of the most commonly used miRNA quantification platforms and has been proven to show better sensitivity and linearity than other platforms (such as the TaqMan Human MicroRNA Array v3.0) in the analysis of plasma samples which often have limited miRNA abundance.^15^, ^24^ However, the results generated from the Exiqon miRNA panel can be inconsistent with the results of qRT‐PCR. Thus, the selected miRNAs were further analyzed among another 36 BC and 36 NC plasma samples using qRT‐PCR. As a result, 11 candidate miRNAs were identified in this two‐step phase and only five miRNAs (let‐7b‐5p, miR‐122‐5p, miR‐146b‐5p, miR‐210‐3p and miR‐215‐5p) were consistently up‐regulated in BC plasma compared to NCs after following multiple‐phase verification using qRT‐PCR.

To evaluate the diagnostic value of the identified miRNAs, we constructed ROC curves and calculated the corresponding AUCs. The AUCs for let‐7b‐5p, miR‐122‐5p, miR‐146b‐5p, miR‐210‐3p and miR‐215‐5p in combined training and testing sets were 0.808, 0.687, 0.623, 0.613 and 0.620, respectively. Evidence has shown that a small panel of miRNAs might possess better predictive value than a single miRNA.^19^ Therefore, we combined the identified miRNAs together and established a 5‐miRNA panel in plasma to better discriminate BC patients from NCs. The AUCs for the panel turned out to be as high as 0.843 for the combined training and testing sets, 0.683 for the training set, 0.966 for the testing set and 0.978 for the external validation set, which demonstrated its better diagnostic performance with higher specificity and sensitivity compared to each individual miRNA biomarker. After strict four‐phase screening, we finally identified a 5‐miRNA signature in plasma which could accurately discriminate BC patients from healthy people.

In this study, during the process of sample grouping and phase division, potential confounding factors were as evenly distributed across the four sets as possible to avoid selection bias. However, epithelial subtype distribution was inevitably unbalanced between the four sets, which might have led to bias in the results. To estimate the confounding effects, we further conducted multiple comparison among BC patients of different epithelial subtypes (luminal, HER2‐enriched, triple‐negative and in situ) based on the expression level of the five identified plasma miRNAs using Kruskal‐Wallis rank test. As a result, no significant difference of miRNA expression was found among the four subgroups, which to some extent reduced the impact of unequal distribution (Table S4).

With increasing research focused on the discovery of circulating miRNAs as tumor biomarkers, the correlation of the five miRNAs identified in our study with BC or other cancer types as well as their multiple biological functions has gradually come to light. Identical to our result, high level of miR‐122‐5p in the blood of BC patients was once reported by Wu et al. Their study revealed that miR‐122‐5p was a potential predictor of BC metastasis in early‐stage patients.^25^ Fong et al provided the explanation that miR‐122 highly secreted by BC cells could promote metastasis by reprogramming glucose metabolism in premetastatic niche.^26^ Moreover, miR‐122‐5p was potential regulator of several BC‐correlated targets such as ADAM10, Syndecan‐1 and IGF1R.^27^, ^28^, ^29^ The diagnostic or prognostic value of circulating miR‐122 was also reported in other cancers such as colorectal cancer and non‐small cell lung cancer, indicating its close connection with tumor biological processes.^30^, ^31^, ^32^ Circulating miR‐215 was previously found to be decreased in plasma in progressive BC patients in comparison with those who did not, but van Schooneveld et al showed just the opposite result of increasing tendency in serum in metastatic BC patients.^33^, ^34^ In BC tissues, down‐regulation of miR‐215‐5p was once reported by Leblanc et al under the effect of its modulator Pax‐5,^35^ and was also observed by Zhou et al which could act as a predictor for pool clinical outcomes for patients.^36^ miR‐215 exhibited not only tumor suppression but also tumor promotion function in some cancer types such as non‐small cell lung cancer, gastric cancer and glioma, which indicated its dual function in cancer development.^37^, ^38^, ^39^, ^40^, ^41^ For miR‐146‐5p, a member of miR‐16 family, it was supposed to be a tumor suppressor for various cancers,^42^, ^43^, ^44^ but little was known about its role in BC. On the contrary, miR‐210 could be a tumor promoter, which was reported to be associated with poor prognosis for BC patients via different biological pathways such as hypoxia induction, invasive transformation and tumor proliferation promotion.^45^, ^46^, ^47^

For better understanding of their biological functions in BC, the expression levels of these identified miRNAs were also explored in 32 pairs of breast tissue samples. Among the five miRNAs, let‐7b‐5p expression in BC tumor tissues was found to be significantly lower in the adjacent normal tissues. Different results of miRNA expression levels between blood and tissue samples have been frequently observed in a number of previous studies.^48^, ^49^ In fact, miRNA expression levels in blood circulation may be quite different from those in tissues.^50^, ^51^ We suspected that this discrepancy might be due to active or passive transport of miRNAs between tumor cells, normal cells adjacent to tumor cells and the tumor microenvironment. Moreover, circulating miRNA expression might be the epitome of systematic disease status, while expression alterations in tissue just reflected local changes. Still, the seemingly conflicting results from some previous studies could give us some hints to the potential role of let‐7b‐5p in BC. Let‐7b‐5p belongs to the let‐7 miRNA family that has been implicated in tumor suppressor activity in various cancers including BC.^52^, ^53^ Lower expression of let‐7b in malignant BC tumor tissue compared to normal tissue or benign BC tumor without lymph node metastasis has also been continuously discovered with considerable evidence.^29^, ^30^, ^35^, ^36^ According to previous studies, the dysregulation of let‐7b‐5p in BC tumor tissues was correlated with tumor‐inhibition activities through different mechanisms such as lowered DNA repair capacity, increased target oncogene expression or disordered inflammatory pathways.^54^, ^55^, ^56^, ^57^ Further studies are required to decipher the exact roles of these miRNAs especially let‐7b‐5p in BC progression and development on this basis.

Furthermore, to decipher the potential forms of these identified miRNAs, we also detected miRNA expression in plasma exosome samples. Exosomes are cell‐derived micro‐vesicles that function in cell‐to‐cell communication within extracellular microenvironment.^58^ Exosomes carry various molecular constituents and miRNAs released from cells are one of the constituents.^50^ The close relationship between exosomal miRNAs and different cancers is being constantly discovered. In this study, among the five identified miRNAs, only miR‐122‐5p was consistently up‐regulated in plasma‐derived exosomes in BC patients. The result was identical to several previous studies.^28^, ^59^ MiR‐122‐5p was enriched in exosomes derived from liver cells.^60^ According to Uen et al, exosomal miR‐122‐5p from liver cells would influence BC mobility by down‐regulating syndecan‐1 (a cell signaling regulator), indicating its potential role in BC metastasis.

Taken together, we proposed a 5‐miRNA signature in plasma which contained let‐7b‐5p, miR‐122‐5p, miR‐146b‐5p, miR‐210‐3p and miR‐215‐5p for BC diagnosis. Surprisingly, none of those miRNA biomarkers once reported by other studies for BC diagnosis were identified in our experiment, like miR‐21, miR‐505, miR‐148b and miR‐210.^61^, ^62^, ^63^ In one of our previous studies, we focused on circulating miRNAs from the miR‐106a‐363 cluster on chromosome X rather than applying the Exiqon miRNA qPCR panel to select candidate miRNAs in the initial screening phase, and identified a 4‐miRNA signature (miR‐106a‐3p, miR‐106a‐5p, miR‐20b‐5p and miR‐92a‐2‐5p) in plasma for BC diagnosis.^21^ However, none of these four plasma miRNAs with significant expression difference between BC patients and NCs were selected in the screening phase in this study. We supposed that the discrepancy between these results might be due to different miRNA lists being screened as well as varied study cohorts or experiment design. The omission of the four miRNAs on chromosome X also revealed the limitation of the miRNA quantification platform. We then revalidated the previous results in the testing set (113 BC vs 113 NCs). The expression of all the four plasma miRNAs showed the same significant difference between BC patients and NCs as described previously (Table S5). Therefore, we further established a 9‐miRNA signature (let‐7b‐5p, miR‐122‐5p, miR‐146b‐5p, miR‐210‐3p, miR‐215‐5p, miR‐106a‐3p, miR‐106a‐5p, miR‐20b‐5p and miR‐92a‐2‐5p) in plasma and constructed the corresponding ROC curves. Diagnostic value of the nine plasma miRNAs was further confirmed in the external validation set (Table S5). The AUCs for the 9‐miRNA panel were 0.968 (95% CI: 0.943‐0.994; Figure S2. (a) for the testing set and 0.989 (95% CI: 0.974‐1.000; Figure S2. (b) for the external validation set. We discovered that compared to the 5‐miRNA panel, the combination of the nine plasma miRNAs showed higher sensitivity and specificity in discriminating BC patients from healthy people. The conclusion could be regarded as an improvement of this study.

In summary, we identified five plasma miRNAs (let‐7b‐5p, miR‐122‐5p, miR‐146b‐5p, miR‐210‐3p, and miR‐215‐5p) by multiple‐phase validation which could serve as novel noninvasive biomarkers for BC diagnosis. However, this study method is far from perfect. For example, the screening list of the Exiqon panel could be incomplete. The results still need to be verified with a larger sample size from multi‐centers. What's more, the underlying association between these miRNAs and BC pathological processes still needs further investigation. Though there is a long way to go before clinical application, we believe that our findings may be useful as a supplement or even replacement for the traditional BC diagnostic strategies in the future.

## DECLARATION

### Ethics approval and consent to participate

Written informed consent was obtained from the patients involved in the study. All the procedures were approved by the Institutional Review Boards of the First Affiliated Hospital of Nanjing Medical University.

## CONFLICT OF INTEREST

The authors declare that they have no conflict of interest.

## Data Availability

The datasets used and analyzed during this study are available from the corresponding author on reasonable request.
