# Supplementary material for: A five‐miRNA panel in plasma was identified for breast cancer diagnosis
Source: Cancer Med. 2019 Sep 30;8(16):7006–17. doi: 10.1002/cam4.2572 (PMC6853814; doi:10.1002/cam4.2572)
Supplement: Supplementary file 1 [file CAM4-8-7006-s001.docx]

**Figure S1.** ROC curve analysis of the five individual miRNAs for BC diagnosis in combined two cohorts of training and testing phases (185 BC VS. 185 NCs). A: let-7b-5p; B: miR-122-5p; C: miR-146b-5p; D: miR-210-3p; E: miR-215-5p. ROC curve: receiver-operating characteristic curve; AUC: area under the ROC curve.


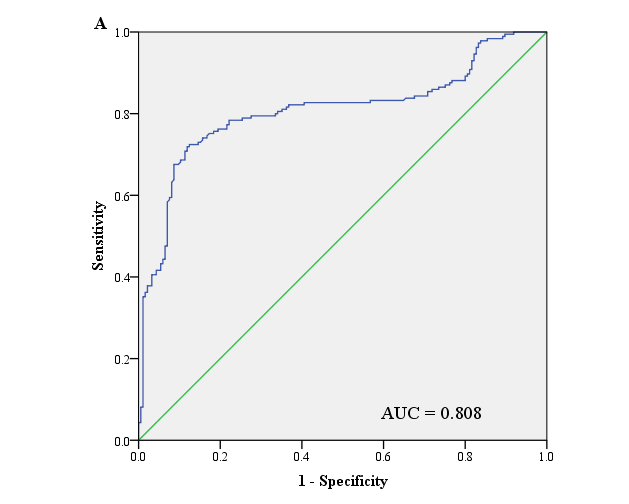

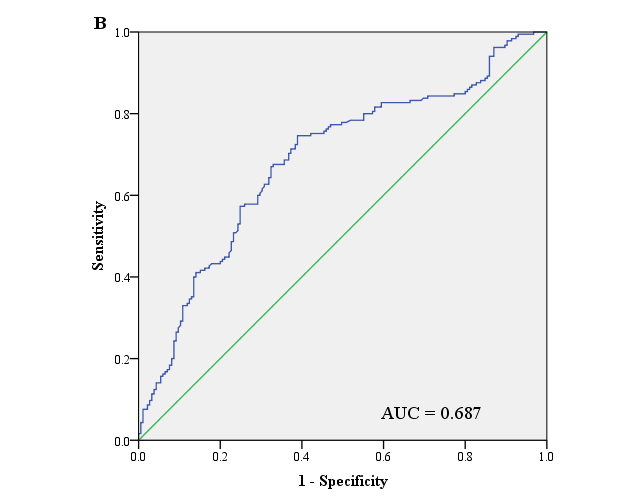

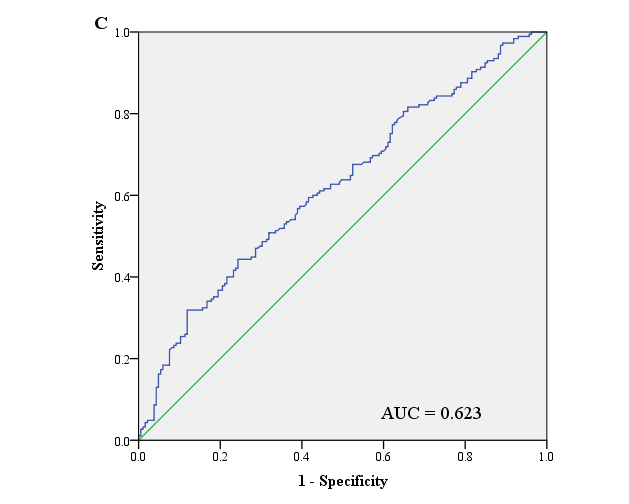

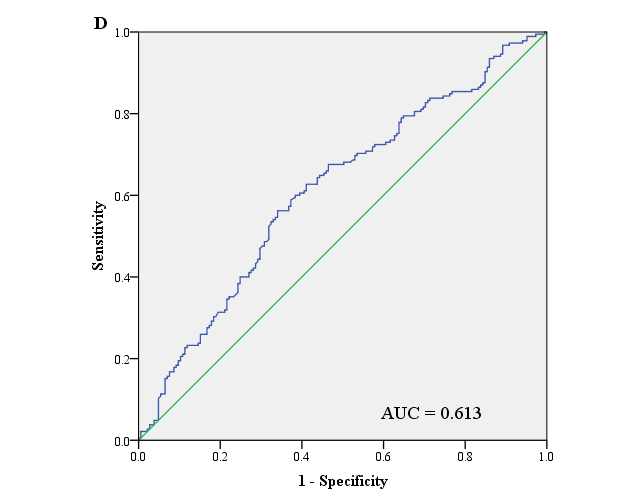

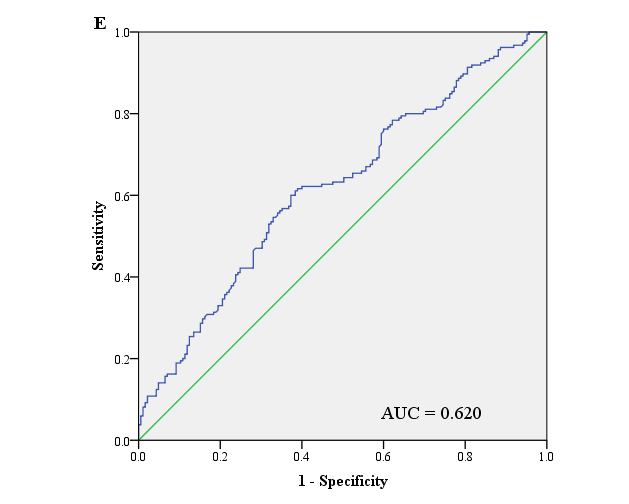


**Figure S2.** ROC curve analysis of the nine-miRNA panel for BC diagnosis. A: testing phase (113 BC VS. 113 NCs); B: external validation phase (36 BC VS. 36 NCs).
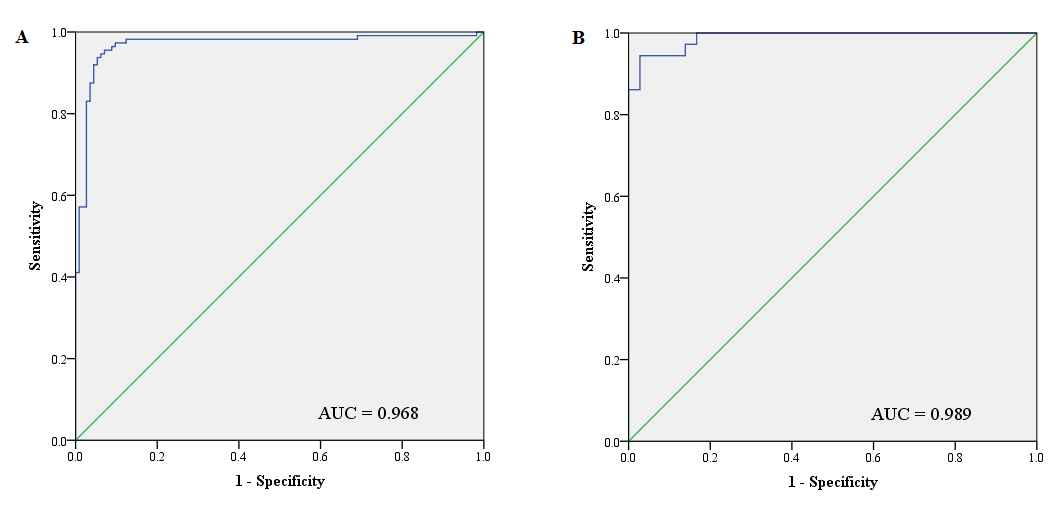


**Table S1.** Differently expressed miRNAs in the screening phase (presented as fold change). Pool 1: randomly mixed group; Pool 2: triple-negative group; Pool 3: HER2-positive group; Pool 4: HR-positive group. Threshold of qRT-PCR: FC > 1.5 or < 0.67, *P* < 0.05.

| **miRNA** | **Exiqon panel** | | | | **qRT-PCR** |
| --- | --- | --- | --- | --- | --- |
|  | **Pool 1** | **Pool 2** | **Pool 3** | **Pool 4** | **36 BC VS. 36 NCs** |
| let-7b-5p | 2.47 |  | 3.60 | 3.34 | **1.62** |
| miR-10b-5p | 2.53 | 1.63 | 3.43 | 3.50 | 1.13 |
| miR-122-5p | 1.76 | 9.44 | 5.67 | 4.88 | **1.93** |
| miR-150-5p | 3.42 |  |  | 1.70 | 0.98 |
| miR-151a-3p | 3.20 |  | 5.91 | 5.25 | **2.70** |
| miR-215-5p | 1.97 |  |  | 1.63 | **3.15** |
| miR-21-5p | 2.26 |  | 1.87 | 1.82 | 0.86 |
| miR-223-5p | 5.60 |  |  |  | **1.82** |
| miR-23a-3p | 2.61 |  | 2.22 | 1.70 | **2.35** |
| miR-23b-3p | 1.99 |  | 2.32 |  | N/A |
| miR-25-3p | 3.45 | 1.65 | 1.73 | 2.57 | 1.02 |
| miR-26a-5p | 1.82 |  | 1.98 |  | N/A |
| miR-375 | 4.93 | 3.85 | 5.38 | 6.56 | 1.36 |
| miR-486-5p | 3.18 |  |  | 1.74 | 0.95 |
| miR-660-5p | 3.75 |  |  |  | **2.65** |
| miR-126-3p |  |  | 1.65 |  | N/A |
| miR-126-5p |  | 3.71 | 6.46 | 4.42 | **1.62** |
| miR-146b-5p |  |  |  | 1.66 | **2.04** |
| miR-195-5p |  |  | 2.08 |  | N/A |
| miR-210-3p | 1.60 |  | 2.53 |  | **2.68** |
| miR-222-3p | 3.77 |  | 1.80 | 1.61 | **1.57** |
| miR-142-3p | -1.72 | -1.99 | -2.21 | -4.70 | N/A |
| miR-144-3p | -6.39 | -1.64 | -8.00 | -3.76 | 1.49 |
| miR-19a-3p | -2.62 | -2.48 | -9.35 | -10.00 | 0.86 |
| miR-19b-3p | -8.23 | -4.24 | -8.51 | -8.62 | 0.91 |
| miR-106b-5p | -1.76 |  | -2.92 | -2.72 | N/A |
| miR-22-3p | -2.84 |  | -3.64 | -3.62 | N/A |
| miR-484 |  | -1.61 | -1.60 |  | 0.85 |
| miR-423-3p |  |  | -2.05 | -4.91 | 0.63 |

**Table S2.** Expression levels of the plasma miRNAs not passing through the selection in the training and testing phases; presented as mean ± SD; ΔCT, relative to combination of cel-miR-39 and miR-16; FC: fold change.

| **miRNA** | **Training phase** | | | | **Testing phase** | | | |
| --- | --- | --- | --- | --- | --- | --- | --- | --- |
|  | **BC** | **HC** | **FC** | **P value** | **BC** | **HC** | **FC** | **P value** |
| **miR-126-5p** | 9.48±  2.56 | 9.38±  2.00 | 0.93 | 0.445 | - | - | - | - |
| **miR-151a-3p** | 6.84±  4.27 | 7.59±  2.52 | 1.68 | 0.846 | - | - | - | - |
| **miR-222-3p** | 8.01±  2.60 | 9.07±  1.98 | 2.09 | 0.028 | 8.44±  1.55 | 8.80± 1.58 | 1.28 | 0.093 |
| **miR-223-5p** | 9.58±  2.24 | 9.83±  1.81 | 1.19 | 0.552 | - | - | - | - |
| **miR-23a-3p** | 6.64±  3.29 | 7.15±  1.80 | 1.42 | 0.908 | - | - | - | - |
| **miR-660-5p** | 5.11± 2.31 | 6.07±  1.03 | 1.94 | 0.048 | 7.37±  0.70 | 7.03± 0.61 | 0.79 | <0.001 |

**Table S3.** Expression levels of the identified five miRNAs in the external validation phase. presented as mean ± SD; ΔCT, relative to combination of cel-miR-39 and miR-16.

| **miRNA** | **BC** | **HC** | **FC** | **P value** | **FDR** |
| --- | --- | --- | --- | --- | --- |
| **let-7b-5p** | 3.81 ± 0.80 | 5.29 ± 0.66 | 2.80 | <0.001 | <0.001 |
| **miR-122-5p** | 4.98 ± 1.08 | 6.00 ± 0.62 | 2.04 | <0.001 | <0.001 |
| **miR-146b-5p** | 9.70 ± 1.25 | 10.74 ± 0.83 | 2.06 | <0.001 | <0.001 |
| **miR-210-3p** | 5.70 ± 0.91 | 6.32 ± 0.54 | 1.54 | 0.004 | 0.0045 |
| **miR-215-5p** | 4.92 ± 2.06 | 5.70 ± 2.19 | 1.72 | 0.033 | 0.0332 |

**Table S4.** Multiple comparison among BC patients of different epithelial subtype (presented as mean ± SD. ΔCT, relative to combination of cel-miR-39 and miR-16; P > 0.05 means there is no reason to reject the null hypothesis that miRNA expression levels are the same among the four subtypes).

|  | **Luminal**  **(n=113)** | **HER2-enriched**  **(n=55)** | **Triple-negative**  **(n=70)** | **In situ**  **(n=19)** | **P-value** |
| --- | --- | --- | --- | --- | --- |
| **let-7b-5p** | 4.02±1.43 | 4.12±1.36 | 4.12±1.33 | 3.83±1.24 | 0.7441 |
| **122-5p** | 4.83±1.79 | 4.82±1.75 | 4.84±1.78 | 4.54±1.91 | 0.9705 |
| **146b-5p** | 9.40±2.11 | 8.98±2.32 | 9.18±2.19 | 8.61±2.75 | 0.5256 |
| **210-3p** | 4.02±2.47 | 4.93±2.69 | 5.07±2.56 | 4.69±2.29 | 0.5319 |
| **215-5p** | 4.02±2.66 | 4.35±2.37 | 4.82±2.60 | 4.26±2.83 | 0.3903 |

**Table S5.** Expression levels of the previously identified four plasma miRNAs in the testing and external validation phase. presented as mean ± SD; ΔCT, relative to combination of cel-miR-39 and miR-16.

| **miRNA** | **Testing phase** | | | | **External validation phase** | | | |
| --- | --- | --- | --- | --- | --- | --- | --- | --- |
|  | **BC** | **HC** | **FC** | **P value** | **BC** | **HC** | **FC** | **P value** |
| **miR-106a-3p** | 8.71  ± 1.72 | 9.61  ± 1.57 | 1.87 | <0.001 | 8.66  ± 1.20 | 9.66  ± 0.89 | 2.01 | 0.001 |
| **miR-106a-5p** | 5.51  ± 0.92 | 6.69  ± 0.95 | 2.26 | <0.001 | 4.46  ± 0.53 | 5.80  ± 0.45 | 2.54 | <0.001 |
| **miR-20b-5p** | 6.27  ± 0.69 | 7.10  ± 0.74 | 1.77 | <0.001 | 5.83  ± 0.53 | 6.66  ± 0.53 | 1.79 | <0.001 |
| **miR-92a-2-5p** | 11.89  ± 1.49 | 12.74  ± 1.26 | 1.80 | <0.001 | 10.57  ± 1.35 | 11.30  ± 1.50 | 1.66 | 0.044 |
